# Targeting the mycobiome in sarcoidosis

**DOI:** 10.3389/fmed.2025.1747685

**Published:** 2026-01-30

**Authors:** Anastasiia Generalova, Yordan Hodzhev, Borislava Tsafarova, Stefan Panaiotov

**Affiliations:** 1 Department of Microbiology, National Center of Infectious and Parasitic Diseases, Sofia, Bulgaria; 2 Department of Natural Sciences, New Bulgarian University, Sofia, Bulgaria

**Keywords:** fungi, granulomatous diseases, microbiome, mycobiome, sarcoidosis

## Abstract

**Introduction:**

Sacoidosis is a multisystem granulomatous disorder characterized by the formation of non-caseating granulomas in affected organs, predominantly the lungs and lymph nodes. Despite extensive research, its etiology remains elusive. Recent evidence suggests fungi may play a role in disease pathogenesis through immune modulation and chronic inflammation.

**Methods:**

We conducted a comprehensive fungal profiling study using samples from thirteen patients with confirmed sarcoidosis and five controls with other pulmonary diseases. Multiple specimen types were analyzed, including bronchoalveolar lavage fluid, lung tissue biopsies, and blood (both cultured and non-cultured). Fungal communities were characterized using ITS (Internal Transcribed Spacer) targeted sequencing followed by bioinformatic analysis.

**Results:**

Distinctive taxonomic patterns emerged in sarcoidosis samples. Several genera previously implicated in the pathogenesis of sarcoidosis were detected, including *Penicillium, Mucor, Saccharomyces*, and *Yarrowia*, which are regarded as potential pathogens. Sample type and processing method significantly influenced community composition, with cultured samples showing reduced diversity dominated by fast-growing taxa.

**Conclusions:**

This study provides evidence of diverse fungal communities in sarcoidosis patients' blood and respiratory specimens, with potential immunomodulatory implications. Our findings integrate with existing epidemiologic and immunologic evidence highlighting fungi as credible antigenic drivers in sarcoidosis and suggest directions for future functional studies exploring fungal-host interactions in disease pathogenesis.

## 1 Introduction

Sarcoidosis is a systemic granulomatous disease of unknown etiology, characterized by the formation of non-caseating granulomas that can impair organ function, most commonly in the lungs and lymph nodes (1–3). Current evidence suggests a multifactorial pathogenesis involving genetic susceptibility, dysregulated immune responses, and environmental or infectious triggers, but the precise mechanisms remain unresolved (2, 3). Corticosteroids are the mainstay of treatment, improving clinical outcomes in many patients, yet their long-term benefits are uncertain and adverse effects are frequent, highlighting the need for alternative therapeutic strategies (4, 5). Other options for steroid-sparing drugs (methotrexate, azathioprine) or anti-TNF therapies (infliximab, adalimumab) for severe cases of sarcoidosis are also applied (6). Sarcoidosis is also manifested as chronic systemic inflammatory disease. In recent years, increasing attention has turned to the role of host-associated microbial communities in chronic inflammatory diseases. While most studies have focused on bacteria, fungi constitute an integral part of the human microbiota and may influence immune homeostasis and disease processes (7). There is evidence that exposure to environmental fungi may influence the development of sarcoidosis (8, 9). The blood mycobiome can be recognized as a potential modulator of systemic inflammation, with fungi and metabolites detectable even in clinically healthy individuals (10). Importantly, fungi are not mere bystanders. Alterations in fungal communities, or fungal dysbiosis, have been associated with immune dysregulation, barrier dysfunction, and chronic inflammatory pathology (7).

The analysis of fungal communities typically relies on internal transcribed spacer (ITS) sequencing or shotgun metagenomics, techniques that allow high-resolution profiling of fungal taxa and their potential functions (11, 12). These approaches have already revealed that fungi are widespread in blood, tissues, and organs previously thought sterile (10). Applying such methods to sarcoidosis could shed light on previously overlooked fungal biodiversity, pathogenic mechanisms, and uncover novel therapeutic targets.

The goal of this research was to study the human fungiome in the blood and respiratory tract, and its potential role in the pathogenesis of sarcoidosis. We further highlight methodological advances for fungiome characterization and discuss how these may be leveraged to explore new avenues for diagnosis and therapy.

## 2 Materials and methods

### 2.1 Ethics statement

Ethical committee approval of the study was issued by Ethical Committee at the Institute of Neurobiology by Bulgarian Academy of Sciences (BAS) decision 38/14.07.2016 and the Institutional Review Board and Institutional Ethics Committee (IRB/IEC) N: 00006384 Protocol No. 2/2025-25.06.2025 at the National Center of Infectious and Parasitic Diseases, and individual written consent were obtained.

### 2.2 Study population

Thirteen patients with confirmed lung sarcoidosis according to standard clinical procedures (13), and from five patients with other pulmonary diseases, including bronchiectasis, chronic inflammatory processes, and other interstitial fibrotic diseases (such as cryptogenic organizing pneumonia). None of the patients had been taking antibiotics or any other medication prior to starting treatment. Blood, lung biopsy and bronchoalveolar lavage samples were collected from the patients. ITS targeted sequencing on DNAs of biopsy, bronchoalveolar lavage, cultured and non-cultured blood samples was applied for biodiversity analysis.

### 2.3 Blood collection and cultivation

Blood was collected in Vacutainer tubes with K3EDTA as anticoagulant (Vacutainer K3E, BD, USA). The blood samples were divided in two parts. One part for culturing (0.5 mL) and another part for direct DNA isolation (0.5 mL). All blood samples were tested for sterility by growing on Sabouraud and blood agar.

Blood culturing was applied in stressful conditions with the aim of resuscitating latent fungi. Two stressful factors were employed: high temperature (cultivation at 43 °C) and toxic stress induced by 1 mg/mL final concentration of menadione sodium bisulfite (vitamin K3). The resuscitation strategy applied was as previously described by Panaiotov et al. (14). Five hundred microliters of blood sample were added to 4.5 mL of Brain Heart Infusion broth (BHI) culture medium (1:10 vol/vol). Culturing was performed in sterile 12 ml polypropylene Falcon tubes (Corning Inc., USA). The sterile culture base medium was composed of Brain Heart Infusion (BHI, Difco, USA) medium and 0.25% sodium citrate. Sterile (D+) sucrose at 10% final concentration and water-soluble form of vitamin K3—menadione sodium bisulfite (Sigma-Aldrich, USA) in a concentration of 1 mg/ml sterilized by filtration were added to the base medium. Resuscitation growth was induced at 43 °C for 24 h. Isolated blood microbiota were confirmed by Gram staining and microscopy. Before DNA extraction, five hundred microliters of whole blood and the whole volume of cultured blood cells were centrifuged at 1,200 g for 20 min, and the pellet was lysed in 10 mL DNase I (1 U/ml) treated, 0.2 μm filtered, and autoclaved dH_2_O. DNase I was added to hydrolyse the cell-free and human DNA. After centrifugation at 1,200 g for 20 min, microbial cells were collected, and the pellet was washed three times with 10 mL dH_2_O. Each wash was followed by centrifugation. The cell pellets were resuspended in 1 mL of lysis buffer containing 500 mM NaCl, 50 mM Tris-HCl, pH 8.0, 50 mM EDTA, 4% sodium dodecyl sulfate, and proteinase K 20 μg/mL. The cell suspension was vigorously homogenized with 0.1/0.3 mm zirconium beads (Biospec Products, Bartlesville, OK, USA) using a bead beater (Benchmark Scientific, Sayreville, NJ, USA) for 3 min at 1,200 g. After microbial cell disintegration, samples were processed for DNA extraction. DNA isolation was performed according to the previously described procedure (14). Isolated fungal blood microbiota DNA was tested by ITS2 PCR analysis with universal primers (15).

### 2.4 Lung biopsy

As part of the obligatory diagnostic procedures, patients suspected of having sarcoidosis underwent transbronchial biopsy, followed by histopathological assessment to detect non-caseating epithelioid-cell granulomas. The specimen measures between 2 and 3 mm in diameter of lung tissue. All patients (mean age ± SD, 48.33 ± 19.14) were positively diagnosed with stage two pulmonary sarcoidosis (13) and included in the study. For DNA extraction, lung tissue samples were resuspended in 1 mL of lysis buffer containing 500 mM NaCl, 50 mM Tris-HCl, pH 8.0, 50 mM EDTA, 4% sodium dodecyl sulfate, and proteinase K 50 μg/mL.

### 2.5 Bronchoalveolar lavage (BAL) samples

Specimens of 20 mL bronchial washings (BAL) with physiological solution (0.9% NaCl) were collected by a clinician in a sterile 50 mL container. No preservative was added. Specimens were transported to the laboratory at 4 °C within a few hours after collection. For DNA extraction BAL samples were centrifuged at 1,200 g for 20 min at 4 °C. The cell pellet was resuspended in 1 mL of lysis buffer containing 500 mM NaCl, 50 mM Tris-HCl, pH 8.0, 50 mM EDTA, 4% sodium dodecyl sulfate, and proteinase K 20 μg/mL.

### 2.6 Controls

Several types of negative controls were included in the analysis: i. simulation of DNA extraction from sterile dH_2_0, including reagents and buffers, ii. DNA extraction from the culture medium, and iii. bronchoscope washouts. Bronchoscope washouts: Ten milliliters of sterile saline solution were used to wash out the sterilized bronchoscopes. There were two types of washout controls: one for sarcoidosis patients and one for patients with other granulomatous diseases. DNA was extracted from each control in the same way as from the clinical samples.

### 2.7 DNA extraction

After performing microbial cell lysis, we isolated the fungal DNA using a modified previously described procedure (16). The modifications included extended, up to 24 h incubation with proteinase K at 51 °C and two centrifugations at 4 °C after the precipitation with 10 M ammonium acetate. As a result, the extracted DNA was purer. Extracted DNA was resuspended in 100 μL 0.1X diluted TE buffer pH 8. The typical yield of DNA was < 100 ng/μL, with a 260/280 nm ratio >1.9.

### 2.8 Sequencing and bioinformatics analysis

High-throughput sequencing and bioinformatics analysis of the samples were carried out by Novogene Co., Ltd. (Beijing, China) following the ITS Amplicon QIIME1 standard workflow. The Internal Transcribed Spacer (ITS) region is the standard DNA barcode for fungi, present in all taxa, variable enough for genus/species-level resolution, and easy to amplify (17, 18). The full ITS (ITS1–5.8S–ITS2) is 500–700 base pairs (bp), which exceeds common Illumina read lengths (150–300 bp) (15). We targeted the highly conserved ITS2 region in fungi using primers with adapters and indices to construct sequencing-ready libraries (17).

Raw sequencing data in FASTQ format were processed using a bioinformatics workflow designed to ensure high-quality sequence assembly, accurate clustering, and reliable taxonomic assignment. Initially, paired-end reads were demultiplexed according to unique sample barcodes, and primer as well as adaptor sequences were trimmed using cutadapt (v3.3). Forward and reverse reads were subsequently merged with FLASH (v1.2.11), producing contiguous raw tags. To ensure data quality, filtering was performed with fastp (v0.23.1), and chimeric sequences were detected and removed by comparison against Unite (ITS) reference databases using vsearch (v2.16.0) tools. The resulting effective tags were then used for downstream analyses. Operational Taxonomic Unit (OTU) clustering was carried out in Uparse (v7.0.1001) at a 97% similarity threshold, with representative sequences from each OTU selected for taxonomic annotation. Fungal ITS regions were classified against the Unite database using BLAST, while unmatched sequences were aligned against the Micro_NT sublibrary of the NCBI NT database. Taxonomic assignment in this latter case was based on the Lowest Common Ancestor (LCA) algorithm, with unclassified lineages excluded to improve annotation accuracy. Phylogenetic relationships among OTUs were inferred by multiple sequence alignment with MUSCLE (v3.8.31). To account for differences in sequencing depth across samples, OTU abundance tables were normalized to the sample with the lowest read count.

### 2.9 Fungal community data processing

A total of 2.06 million ITS reads were obtained across all samples, with an average of approximately 31,000 reads per sample (Supplementary Table 1). Amplicon sequence tables were analyzed at multiple taxonomic levels using in-house Python scripts. For descriptive community summaries, relative abundances (%) were calculated within each sample and subsequently averaged across grouped materials defined by clinical origin, according to a predefined sample–material mapping:

S-BAL—bronchoalveolar lavage sample obtained from a patient with sarcoidosis; S-biopsy—lung tissue biopsy sample from a patient with sarcoidosis; S-control—negative control sample associated with sarcoidosis cases; S-cultured—cultured blood sample obtained from a patient with sarcoidosis; S-non-cultured—non-cultured blood sample obtained from a patient with sarcoidosis; S-washout—negative control, bronchoscope washout sample; P-BAL—bronchoalveolar lavage sample obtained from a patient with another pulmonary disease; P-biopsy—lung tissue biopsy sample from a patient with another pulmonary disease; P-blood—peripheral blood sample from a patient with another pulmonary disease; P-control—negative control sample associated with patients with other pulmonary diseases; P-washout—negative control, bronchoscope washout sample.

To minimize the impact of spurious detections and low-level environmental DNA, taxa with fewer than 100 total reads across all samples were excluded. Within each sample, a taxon was considered present only if its read count reached at least 0.1% of the sample's total reads. For the main analyses starting from the order level, taxa detected in any control or washout samples were removed prior to summarizing the mean numbers of detected taxa per material and identifying unique taxa, except for blood samples, which were filtered only against the controls. All data processing and summarization were performed in Python 3.11 using the pandas and numpy libraries.

## 3 Results

### 3.1 Fungal community composition at the phylum level

Across all analyzed materials, the fungal community was dominated by members of the *Ascomycota* and *Basidiomycota*, with minor contributions from early-diverging fungal lineages and unclassified taxa. The relative proportions of the dominant phyla varied substantially between sample groups.

Among the samples from patients with other granulomatous diseases (P-series), *Ascomycota* represented 21.2–96.0% of total fungal reads, reaching the highest proportion in P-biopsy (96.0%) and P-control (91.9%), whereas *Basidiomycota* accounted for 3.0–65.1%, peaking in P-BAL (65.1%). Unclassified or rare phyla together comprised up to 24.6% in P-washout, mainly represented by *Chytridiomycota, Rozellomycota*, and *Mortierellomycota*.

Among the samples from patients with sarcoidosis (S-series), *Ascomycota* similarly dominated most materials, contributing 56.9–70.1% of total reads, while *Basidiomycota* accounted for 9.9–28.4%, with the highest share in S-biopsy (28.4%) and S-BAL (23.5%). Minor phyla such as *Rozellomycota, Mortierellomycota*, and *Mucoromycota* together represented less than 10% in most sample types but reached up to 6.4% in S-non-cultured and 5.0% in S-control samples.

Notably, *Zoopagomycota* sequences were primarily detected in S-cultured samples (13.7%), reflecting the broader diversity captured in cultured materials. Overall, the data indicate that *Ascomycota* and *Basidiomycota* jointly dominated all materials, while early-diverging and unclassified fungal lineages contributed variably across groups, particularly in washout and cultured samples, likely reflecting environmental or low-abundance taxa.

### 3.2 Description of OTU counts at the order level

Table 1 summarizes the mean number of operational taxonomic units (OTUs) identified at the order level, grouped according to the type of biological material analyzed.

**Table 1:** Mean (±SD) number of orders identified across sample groups.

**Group**	**No. of samples**	**Mean**	**SD**
P-BAL	5	17.6	8.08
P-biopsy	4	12	5.94
P-blood	5	16.4	5.03
P-control	1	8	
P-washout	4	23.25	15.52
S-BAL	12	20.33	7.39
S-biopsy	13	27.62	17.41
S-control	5	6.6	2.3
S-cultured	11	16.64	15.1
S-non-cultured	15	23.87	17.25
S-washout	9	22.56	19.16

#### 3.2.1 Unique taxa analysis

To prove unique taxonomic orders, orders detected in control or washout samples (both S and P series) were excluded from further comparisons. The remaining taxa were then assessed for their occurrence across all sample–material groups (e.g., S-BAL, P-biopsy, etc.). A taxon was considered unique if it was detected in at least one sample of a single group and was absent from all other groups. Using this conservative definition, only three unique fungal orders were identified across the dataset: environmental *Paraglomerales* and *Branch06* (unclassified order in *Sordariomycetes*) were found exclusively in S-BAL samples, while *Myrmecridiales* was detected only in S-non-cultured material. These orders appeared with total read counts of 442, 320, and 167, respectively.

The taxonomic composition was summarized by identifying the three most abundant orders within each sample-material group based on relative read abundance. The majority of the groups were dominated by one or two taxa contributing over 60% of total reads, reflecting a highly uneven community structure (Table 2).

**Table 2:** Top-3 most abundant fungal orders.

**Group**	**Top 1**	**Top 1 percent**	**Top 2**	**Top 2 percent**	**Top 3**	**Top 3 percent**
P-BAL	*Zoopagomycota_ord_Incertae_sedis*	58.88	*Geminibasidiales*	31.11	GS05	8.96
P-biopsy	*Trechisporales*	99.92	*Pertusariales*	0.08	–	–
P-blood	*Venturiales*	19.83	*Filobasidiales*	17.15	*Tremellales*	17.06
S-BAL	*Trechisporales*	42.75	*Magnaporthales*	28.21	*Paraglomerales*	10.18
S-biopsy	*Trechisporales*	33.1	*Pucciniales*	15.29	*Sebacinales*	11.25
S-cultured	*Zoopagomycota_ord_Incertae_sedis*	74.04	*Tremellales*	5.88	*Rozellomycota_ord_Incertae_sedis*	4.30
S-non-cultured	*Pezizomycotina_ord_Incertae_sedis*	28.19	*Mycosphaerellales*	22.85	*Tremellales*	13.31

Among samples from patients without sarcoidosis (P-series), *Zoopagomycota ord. Incertae sedis* was the most abundant order in P-BAL (58.9%), followed by *Geminisbasidiales* (31.1%) and *GS05* (9.0%). P-biopsy samples were overwhelmingly dominated by *Trechisporales* (99.9%), while P-blood was characterized by a co-dominance of *Venturiales* (19.83%), *Filobasidiales* (17.15%) and *Tremellales* (17.06%).

In the sarcoidosis (S-series) cohort, *Trechisporales* predominated in both S-BAL (42.8%) and S-biopsy (33.1%) samples, together with *Magnaporthales* and *Pucciniales*, respectively. S-cultured material was dominated by *Zoopagomycota ord. Incertae sedis* (74.04%), while S-non-cultured samples were enriched in *Pezizomycotina ord. Incertae sedis* (28.19%), *Mycosphaerellales* (22.85%) and *Tremellales* (13.31%). Across all groups, the cumulative contribution of the top three orders exceeded 85% of the total reads, indicating that only a few taxa accounted for the majority of fungal community abundance in each material.

### 3.3 Family-level diversity and dominant taxa

At the family level, the number of detected taxa varied substantially across materials and disease groups. In non-sarcoidosis samples (P-series), the mean number of families per group ranged from 12.3 ± 5.4 in P-biopsy to 32.5 ± 25.6 in P-washout, while in sarcoidosis samples (S-series) it ranged from 7.4 ± 1.7 in S-control to 37.9 ± 30.9 in S-biopsy. Overall, BAL and non-cultured materials tended to harbor the richest communities (~25–32 families on average), whereas control samples consistently showed the lowest diversity (< 10 families per sample) (Table 3).

**Table 3:** Mean (±SD) number of families identified across sample groups.

**Group**	***N***	**Mean**	**SD**
P-BAL	5	22.8	13.75
P-biopsy	4	12.25	5.38
P-blood	5	19.6	11.89
P-control	1	10	
P-washout	4	32.5	25.63
S-BAL	12	24.92	11.01
S-biopsy	13	37.92	30.89
S-control	5	7.4	1.67
S-cultured	11	22.64	25.55
S-non-cultured	15	31.87	29.69
S-washout	9	31.11	30.6

Across sample-material groups, fungal communities were dominated by a limited number of families (Table 4). In the non-sarcoidosis cohort, *Trichomeriaceae* (30.5%), *Zoopagomycota fam. Incertae sedis* (22.8%) and *Physalacriaceae* (12.2%) prevailed in P-BAL samples; *Hydnodontaceae* (64.0%) and *Dissoconiaceae* (30.5%) dominated P-biopsy material; while *Hypocreales fam. Incertae sedis* (11.76%), *Venturiaceae* (10.42%), and *Rhizophydiales fam. Incertae sedis* (8.71%) were characteristic of P-blood.

**Table 4:** Top-3 most abundant fungal families.

**Group**	**Top 1**	**Top 1 percent**	**Top 2**	**Top 2 percent**	**Top 3**	**Top 3 percent**
P-BAL	*Trichomeriaceae*	30.48	*Zoopagomycota_fam_Incertae_sedis*	22.79	*Physalacriaceae*	12.18
P-biopsy	*Hydnodontaceae*	64.01	*Dissoconiaceae*	30.45	*Hydnaceae*	2.87
P-blood	*Hypocreales_fam_Incertae_sedis*	11.76	*Venturiaceae*	10.42	*Rhizophydiales_fam_Incertae_sedis*	8.71
S-BAL	*Mucoraceae*	20.39	*Incrustoporiaceae*	18.41	*Phanerochaetaceae*	16.26
S-biopsy	*Botryobasidiaceae*	21.5	*Psathyrellaceae*	18.73	*Schizoporaceae*	9.77
S-cultured	*Zoopagomycota_fam_Incertae_sedis*	42.99	*Saccharomycetales_fam_Incertae_sedis*	13.58	*Cucurbitariaceae*	9.56
S-non-cultured	*Pezizomycotina_fam_Incertae_sedis*	23.93	*Teratosphaeriaceae*	17.71	*Rozellomycota_fam_Incertae_sedis*	8.72

In sarcoidosis samples, *Mucoraceae, Incrustoporiaceae*, and *Phanerochaetaceae* were the most abundant in S-BAL (20.4%, 18.4%, 16.3%), and *Botryobasidiaceae, Psathyrellaceae*, and *Schizoporaceae* dominated S-biopsy. S-cultured samples were almost entirely dominated by *Zoopagomycota fam. Incertae sedis* (42.99%), whereas S-non-cultured samples were characterized by *Pezizomycotina fam. Incertae sedis* (23.93%), *Teratosphaeriaceae* (17.71%), and *Rozellomycota fam. Incertae sedis* (8.72%). In most groups, the cumulative relative abundance of the top three families exceeded 85%, indicating pronounced community unevenness.

After excluding family taxa detected in control and washout samples, a small number of unique families remained specific to certain sarcoidosis materials. Notably, *Magnaporthaceae, Branch06 fam. Incertae sedis, Pervetustaceae*, and *Tubulicrinaceae* were restricted to S-BAL samples, *Botryobasidiaceae* and *Sirobasidiaceae* to S-biopsy, and *Myrmecridiaceae* and *Zoopagales fam. Incertae sedis* to S-non-cultured samples. Among non-sarcoidosis samples, *Reticulascaceae* (P-blood) and *Saccharomycodaceae* (P-BAL) were found exclusively in their respective materials. These unique families accounted for a very small proportion of total reads, highlighting the predominance of shared fungal lineages across both cohorts (Table 5).

**Table 5:** Unique fungal families detected exclusively in sample–material groups.

**Group**	**Family**	**No. of samples**	**Total reads in group**
S-BAL	*Magnaporthaceae*	1	1,225
S-BAL	*Hymenochaetaceae*	2	633
S-BAL	*Tubulicrinaceae*	1	580
S-BAL	*Pervetustaceae*	1	442
S-BAL	*Branch06_fam_Incertae_sedis*	1	320
S-BAL	*Steccherinaceae*	2	312
S-BAL	*Amphisphaeriales_fam_Incertae_sedis*	1	182
S-BAL	*Auriscalpiaceae*	1	125
S-BAL	*Dermateaceae*	1	114
S-biopsy	*Botryobasidiaceae*	2	1,851
S-biopsy	*Sirobasidiaceae*	1	412
S-biopsy	*Exidiaceae*	2	120
S-biopsy	*Claroideoglomeraceae*	1	115
S-cultured	*Agaricostilbaceae*	1	400
S-non-cultured	*Zoopagales_fam_Incertae_sedis*	1	226
S-non-cultured	*Myrmecridiaceae*	1	167
P-blood	*Reticulascaceae*	1	494
P-BAL	*Saccharomycodaceae*	1	100

#### 3.3.1 MicrobiomeAnalyst visualization

Fungal family-level profiles were examined using MicrobiomeAnalyst (19), a user-friendly, web-based platform that enables researchers and clinicians without extensive programming expertise to perform comprehensive microbiome data processing and statistical analyses (20, 21). The feature abundance table, containing raw count data derived from the OTU abundance table in text format, was used as the primary input. Sample identifiers were fully matched between the metadata and OTU tables, resulting in a total of 83 samples included in the analysis. P-control as a single sample in the group was not added as it is not enough for comparisons.

Following initial quality control, 426 OTUs were retained from 1515 original entries. Singleton features, appearing in only one sample, were removed as potential sequencing artifacts. Additional filtering was applied to improve the robustness of downstream analyses: features with fewer than four counts in less than 20% of samples or with low variability (interquartile range < 10%) were excluded.

Normalization was subsequently performed to account for differences in sequencing depth and data sparsity, ensuring biologically meaningful comparisons across samples. These steps were implemented automatically by MicrobiomeAnalyst using the filtered count data.

In total, the final dataset comprised 83 matched samples and 21 high-quality OTUs after filtering and normalization. The processed data were used for all subsequent statistical analyses and graphical visualizations.

**Figure 1:**
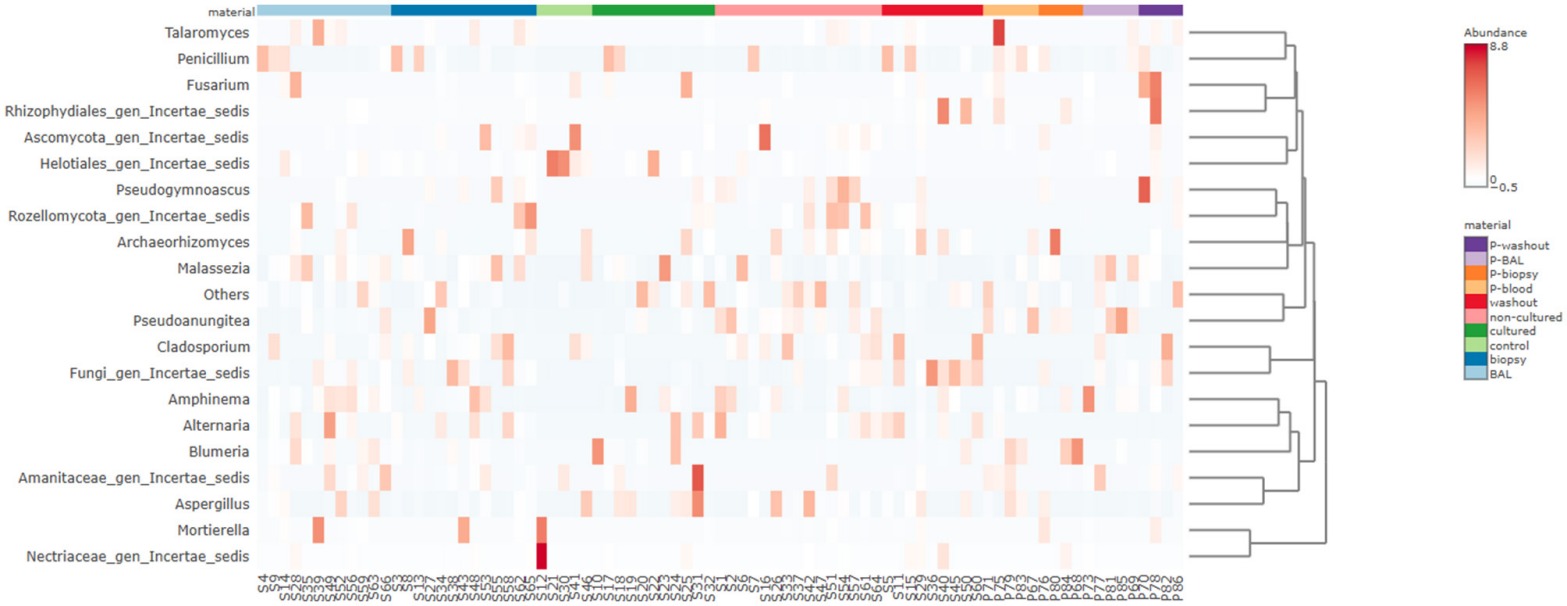
Heatmap diagram of fungal microbiota composition at family level of corresponding experimental materials. The rows represent taxonomic groups at the family level, while the columns correspond to the experimental materials, including BAL, biopsy, control, cultured, non-cultured, washout, and non-sarcoidosis-patient-derived samples (P-BAL, P-biopsy, P-blood, P-washout). Color intensity reflects the standardized abundance values, with red indicating higher abundance and blue representing lower abundance.

A heatmap (Figure 1) was generated in MicrobiomeAnalyst to visualize the relative abundance patterns of dominant fungal taxa across different sample types. Hierarchical clustering was applied both to taxa and samples using Euclidean distance and Ward's linkage method, allowing the identification of similarity patterns among microbial communities.

**Figure 2:**
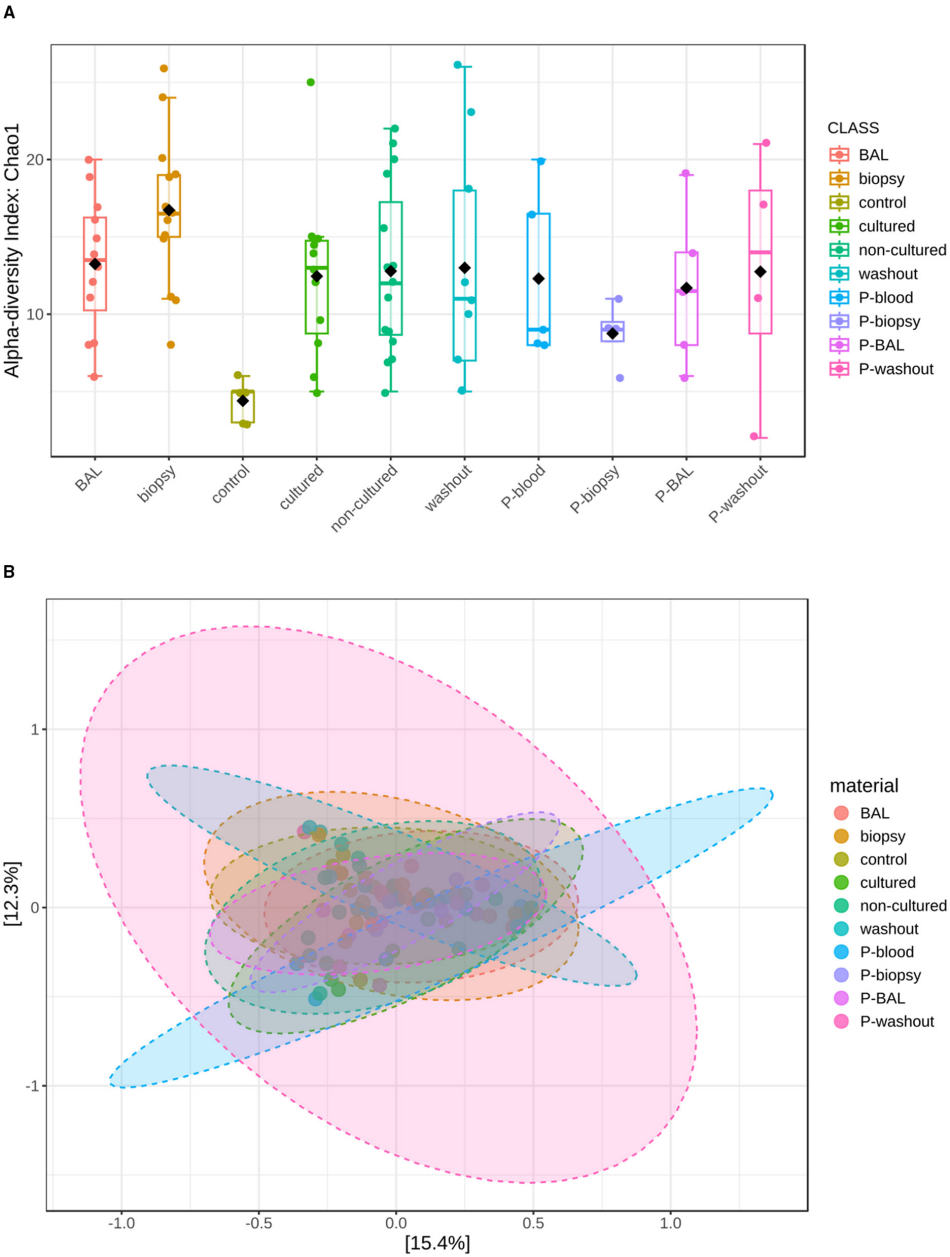
Community diversity analysis for mycobiota at family level. **(A)** Represents Alpha-diversity (Chao 1 index) measuring fungal richness. **(B)** Shows fungal beta-diversity distance between samples in two dimensions, with individual points representing OTU distribution of each sample based on the principal coordinates analysis (PcoA).

Alpha diversity (Figure 2A), assessed by the Chao1 index, revealed significant differences between several sample groups (S-biopsy, S-BAL, S-cultured, S-non-cultured) and the S-control after FDR correction (FDR < 0.05), as well as between P-biopsy and S-biopsy (FDR = 0.0143). Beta diversity (Figure 2B), based on Bray–Curtis dissimilarity and visualized by PCoA, differed significantly among sample types (PERMANOVA: *F*-value: 1.8133; *R*-squared: 0.18; *p*-value: 0.001).

Specifically, microbial profiles of biopsy (*F* = 2.49, FDR = 0.027), non-cultured (*F* = 3.46, FDR = 0.027), BAL (*F* = 3.39, FDR = 0.0225), and washout samples (*F* = 3.61, FDR = 0.027) were all significantly distinct from controls. Furthermore, non-cultured samples differed from washout specimens (*F* = 3.63, FDR = 0.0225), and cultured samples displayed significant compositional shifts relative to washout samples (*F* = 2.67, FDR = 0.04), suggesting that both sampling method and cultivation influence community structure. These results highlight pronounced microbial differentiation between tissue-associated and lavage-derived communities.

### 3.4 Genus-level diversity

At the genus level, the richness of fungal taxa varied widely between materials and disease groups. In the non-sarcoidosis cohort (P-series), the mean number of genera per group ranged from 12.5 ± 5.8 in P-biopsy to 34.0 ± 28.9 in P-washout. In sarcoidosis samples (S-series), genus richness ranged from 7.4 ± 1.7 in S-control to 40.6 ± 36.7 in S-biopsy. Overall, BAL and non-cultured materials exhibited relatively high genus diversity (~25–33 genera per sample), while control materials consistently displayed the lowest richness (< 10 genera) (Table 6).

**Table 6:** Mean (±SD) number of genera identified across sample groups.

**Group**	***N***	**Mean**	**SD**
P-BAL	5	24.2	15.12
P-biopsy	4	12.5	5.8
P-blood	5	21.2	13.79
P-control	1	10	
P-washout	4	34	28.88
S-BAL	12	25.58	10.93
S-biopsy	13	40.62	36.69
S-control	5	7.4	1.67
S-cultured	11	24.45	28.13
S-non-cultured	15	33.2	34.47
S-washout	9	35.33	36.9

Community composition at the genus level in all tested materials was dominated by a few highly abundant taxa (Table 7).

**Table 7:** Top-3 most abundant fungal genera.

**Group**	**Top 1**	**Top 1 percent**	**Top 2**	**Top 2 percent**	**Top 3**	**Top 3 percent**
P-BAL	*Rhodotorula*	26.46	*Trichomeriaceae_gen_Incertae_sedis*	18.38	*Zoopagomycota_gen_Incertae_sedis*	14.39
P-biopsy	*Trechispora*	60.9	*Dissoconium*	28.92	*Thelephora*	5.56
P-blood	*Penicillium*	37.29	*Talaromyces*	35.15	*Pseudoanungitea*	2.85
S-BAL	*Neophaeococcomyces*	26.27	*Amanita*	12.22	*Mucor*	9.66
S-biopsy	*Yarrowia*	21.99	*Botryobasidium*	11.34	*Sphaerographium*	9.61
S-cultured	*Saccharomyces*	29.66	*Zoopagomycota_gen_Incertae_sedis*	20.57	*Penicillium*	18.23
S-non-cultured	*Hormiactis*	23.05	*Salinomyces*	16.51	*Rozellomycota_gen_Incertae_sedis*	8.50

In non-sarcoidosis samples, *Rhodotorula* (26.5%), *Trichomeriaceae* gen. Incertae sedis (18.4%), and *Zoopagomycota gen. Incertae sedis* (14.4%) predominated in P-BAL; *Trechispora* (60.9%) and *Dissoconium* (28.9%) in P-biopsy; and Penicillium, *Talaromyces*, and *Pseudoanungitea* (37.29%, 35.15%, 2.85%) in P-blood.

In sarcoidosis samples, *Neophaeococcomyces* (26.3%), *Amanita* (12.2%), and *Mucor* (9.7%) were most abundant in S-BAL; *Yarrowia* (22.0%), *Botryobasidium* (11.3%), and *Sphaerographium* (9.6%) dominated S-biopsy; while *Saccharomyces* (29.66%), *Zoopagomycota gen. Incertae sedis* (20.57%) and *Penicillium* (18.23%) were characteristic of S-cultured samples. S-non-cultured samples were dominated by *Hormiactis* (23.05%) and *Salinomyces* (16.51%). In all groups, the cumulative relative abundance of the three dominant genera exceeded 80% of total reads, indicating a strong community unevenness.

After exclusion of taxa detected in control and washout samples, several genera remained unique to specific sarcoidosis materials. In S-BAL, unique taxa included *Amanita, Magnaporthe, Dactylonectria, Subulicystidium, Phlebiopsis, Pervetustaceae gen. Incertae sedis, Branch06 gen. Incertae sedis*, and *Sarcostroma*. In *S-biopsy, Botryobasidium, Schizoporaceae gen. Incertae sedis*, and *Muriphaeosphaeria* were unique, whereas S-cultured samples contained *Sterigmatomyces, Cylindrocladiella*, and *Cyberlindnera*. S-non-cultured materials showed exclusive presence of *Phanerochaete, Zoopagales gen. Incertae sedis*, and *Myrmecridium*. In the non-sarcoidosis cohort, *Lectera, Arnium*, and *Reticulascus* were unique to P-blood, while *Trichomeriaceae gen. Incertae sedis* and *Cutaneotrichosporon* were exclusive to P-BAL. These genera occurred at low absolute read counts (< 1 × 103 per group) and likely represent rare or condition-specific taxa (Table 8).

**Table 8:** Unique fungal genera detected exclusively in sample–material groups.

**Group**	**Order**	**No. of samples**	**Total reads in group**
S-BAL	*Amanita*	1	6,600
S-BAL	*Magnaporthe*	1	1,225
S-BAL	*Dactylonectria*	1	1,054
S-BAL	*Subulicystidium*	1	662
S-BAL	*Fuscoporia*	1	620
S-BAL	*Tubulicrinis*	1	580
S-BAL	*Phlebiopsis*	1	453
S-BAL	*Pervetustaceae_gen_Incertae_sedis*	1	442
S-BAL	*Sarcostroma*	1	440
S-BAL	*Branch06_gen_Incertae_sedis*	1	320
S-BAL	*Issatchenkia*	1	249
S-BAL	*Entyloma*	1	244
S-BAL	*Simplicillium*	1	227
S-BAL	*Antrodiella*	1	218
S-BAL	*Ascochyta*	1	217
S-BAL	*Amphisphaeriales_gen_Incertae_sedis*	1	182
S-BAL	*Collybia*	1	122
S-BAL	*Auriscalpium*	1	121
S-BAL	*Naevala*	1	114
S-biopsy	*Botryobasidium*	1	1,848
S-biopsy	*Schizoporaceae_gen_Incertae_sedis*	1	587
S-biopsy	*Muriphaeosphaeria*	1	460
S-biopsy	*Fibulobasidium*	1	412
S-biopsy	*Pseudoteratosphaeria*	1	173
S-biopsy	*Claroideoglomus*	1	115
S-cultured	*Sterigmatomyces*	1	400
S-cultured	*Cylindrocladiella*	1	334
S-cultured	*Cyberlindnera*	1	284
S-cultured	*Clavaria*	1	208
S-cultured	*Xeromyces*	1	188
S-non-cultured	*Phanerochaete*	2	276
S-non-cultured	*Zoopagales_gen_Incertae_sedis*	1	226
S-non-cultured	*Myrmecridium*	1	167
S-non-cultured	*Bullera*	2	131
P-blood	*Lectera*	1	732
P-blood	*Arnium*	1	624
P-blood	*Reticulascus*	1	494
P-biopsy	*Thelephora*	1	118
P-BAL	*Trichomeriaceae_gen_Incertae_sedis*	1	428
P-BAL	*Cutaneotrichosporon*	1	213
P-BAL	*Flammulina*	1	179
P-BAL	*Hanseniaspora*	1	100

Several genera typically associated with macrofungi (e.g., *Amanita, Clavaria, Phlebiopsis, Collybia*) were detected in low abundance within both sarcoidosis and non-sarcoidosis samples. These genera are not expected to be active colonizers of human tissues; instead, their detection likely reflects environmental DNA originating from fungal spores, hyphal fragments, or extracellular material. The presence of macromycete sequences in respiratory samples is consistent with the high airborne spore load of *Basidiomycota* during much of the year, especially in temperate climates, where spores of *Amanita, Boletus, Clavaria* and related taxa are common components of bioaerosols.

Alternatively, short DNA fragments from decayed plant material or soil particles introduced during sampling or sample handling may also contribute to this background signal. Because these taxa were generally represented by very low read counts and were not confined to a specific disease group, they are most plausibly interpreted as environmental or technical contaminants rather than indicators of active infection. Nevertheless, their recurrent detection highlights the sensitivity of amplicon sequencing for detecting trace environmental fungal DNA, even in clinical materials that are not expected to harbor viable macromycetes.

Among the genera that appeared recurrently across the analyzed materials, several taxa with potential clinical relevance were detected, including *Neophaeococcomyces, Yarrowia, Mucor*, and *Saccharomyces*. Although these genera encompass species known as opportunistic or environmental fungi, their presence in the present dataset was characterized by low abundance and limited group specificity. *Mucor*, a genus that includes recognized agents of mucormycosis, was observed sporadically and at low read counts, suggesting that it represents background DNA rather than evidence of active infection. *Yarrowia*, typically associated with industrial environments and occasionally isolated from clinical specimens, is regarded as an opportunistic colonizer rather than a primary pathogen; its detection here is likely incidental. Similarly, *Saccharomyces*, commonly encountered in food and fermentation processes, is an infrequent cause of infection and probably reflects dietary or environmental DNA contamination. *Neophaeococcomyces*, primarily known from plant and soil habitats, also likely represents environmental or airborne fungal material introduced during sampling and processing. While these findings are not diagnostic, they may inform future targeted studies aiming to distinguish true colonizers from background fungal DNA in respiratory or tissue samples.

When compared to the filtered analysis where control and washout samples were excluded and low-abundance taxa were removed, the unfiltered genus-level profiles provide a useful view of the broader environmental background signal in these materials. The dominance of ubiquitous molds (*Penicillium, Talaromyces, Cladosporium*) and surface-associated yeasts (*Candida, Malassezia*) in the raw dataset highlights the substantial contribution of airborne spores, laboratory- or reagent-associated DNA, and transient environmental contaminants to the observed mycobiome signal (Table 9).

**Table 9:** Top-3 most abundant fungal genera including controls.

**Group**	**Top 1**	**Top 1 percent**	**Top 2**	**top 2 percent**	**Top 3**	**Top 3 percent**
P-BAL	*Amphinema*	54.82	*Malassezia*	7.88	Others^*^	7.73
P-biopsy	*Blumeria*	54.72	*Discosia*	36.44	*Amphinema*	1.4
P-blood	*Penicillium*	28.13	*Talaromyces*	26.52	Others	5.37
P-control	*Thermomyces*	30.04	*Nigrospora*	26.46	*Dipodascaceae* gen. *Incertae sedis*	21.38
P-washout	Others	15.5	*Fungi* gen. *Incertae sedis*	9.02	*Cladosporium*	6.64
S-BAL	*Candida*	12.9	*Penicillium*	12.26	*Amphinema*	6.58
S-biopsy	*Malassezia*	11.79	*Salinomyces*	7.66	*Cladosporium*	7.49
S-control	*Mycena*	8.85	*Botryotrichum*	8.15	*Oidiodendron*	7.69
S-cultured	*Saccharomyces*	19.74	*Zoopagomycota* gen. *Incertae sedis*	13.69	*Penicillium*	12.14
S-non-cultured	*Hormiactis*	17.45	*Salinomyces*	12.5	*Rozellomycota* gen. *Incertae sedis*	6.44
S-washout	*Penicillium*	18.17	*Fungi* gen. *Incertae sedis*	13.25	*Nigrospora*	10.63

^*^Others here and below refers to a taxon's name obtained through sequencing and bioinformatics analysis, rather than a collection of taxa excluding those already listed.

## 4 Discussion

Our multi-matrix mycobiome profiling recovered several fungal taxa with prior evidence linking fungi to sarcoidosis, and it helps position specific genera within that broader framework.

We detected *Penicillium* at relatively high abundance in washout and BAL specimens (12%). *Penicillium* species are widely diffused in air, water and food and the genus includes more than 300 species (22). Part of them are well-known producers of highly toxic mycotoxins. Prior clinical reports describe positive serum precipitins to *Penicillium* in patients who simultaneously fulfilled criteria for sarcoidosis and hypersensitivity pneumonitis, implying that *Penicillium* exposure can participate in granulomatous immune activation in at least a subset of cases (23). Cases of chronic granulomatous disorder (CGD) with pulmonary infection caused by *Penicillium spp*. have been described in the literature (24, 25). In our data, the prominence of *Penicillium* in the airway lumen aligns with inhalational exposure as a plausible route (23).

*Candida* was among the taxa enriched in BAL. Independent immunologic studies have documented elevated antifungal antibodies against *Candida albicans* (β-glucan and mannan antigens) in both BAL and serum of sarcoidosis patients, indicating heightened immune recognition of *Candida* components (26). Likewise, *Saccharomyces* antigens elicited increased antifungal antibody responses in sarcoidosis cohorts, further supporting a role for yeast-derived cell-wall moieties in ongoing immune stimulation (26).

We found genera that are major sources of β-glucan (e.g., *Penicillium, Alternaria, Cladosporium*), and earlier studies have identified β-glucan in sarcoidosis BAL and mediastinal lymph nodes, where its quantity correlated with the extent of parenchymal granulomas and nodal enlargement (27). These observations provide a biologically coherent link between environmental molds, their conserved cell-wall polymers, and granulomatous inflammation (27).

Notably, *Alternaria* and *Cladosporium* were abundant in washout samples, a pattern compatible with external environmental carriage or superficial colonization. Epidemiologic studies show that patients with active or recurrent sarcoidosis more often reside in homes with higher fungal presence, supporting a contribution of indoor mold exposure to disease risk. Such studies specifically implicate “moldy” environments rather than a single species (8), but *Alternaria* and *Cladosporium* are canonical indoor/outdoor molds in these settings. These taxa in our washouts therefore fit the exposure model rather than deep tissue residency (8).

*Malassezia* was enriched in both BAL and biopsy specimens in our dataset. While *Malassezia* is a common commensal of human skin and mucosal surfaces, and not traditionally considered a sarcoid pathogen, its presence in tissue-associated samples is intriguing. Previous literature has highlighted that *Malassezia* metabolites are implicated in seborrheic dermatitis, and clinical cases have shown that seborrheic dermatitis driven by *Malassezia* may mimic cutaneous sarcoidosis, leading to diagnostic confusion (28). Thus, although a direct etiological link between *Malassezia* and sarcoidosis has not been established in the pulmonary context, the potential for this genus to trigger granulomatous-like responses or to obscure clinical diagnosis warrants further investigation.

Taken together, our findings integrate with epidemiologic, immunologic, and mechanistic lines of evidence to highlight fungi as credible antigenic drivers in sarcoidosis. Main clinically relevant fungi detected are listed in Table 10.

**Table 10:** Clinically relevant fungal genera detected across sample types.

**Genus**	**Clinical relevance**	**Approx. relative abundance (range)**
*Penicillium*	Environmental mold; β-glucan source; associated with hypersensitivity and granulomatous responses (22–24, 26)	37.3%—P-blood
*Mucor*	Opportunistic molds that can colonize the airways of patients with chronic lung disease and cause severe pulmonary mucormycosis (30, 31)	9.7%—S-BAL
*Yarrowia*	Opportunistic fungus capable of causing bloodstream, nail, and occasional pulmonary infections, supported by its demonstrated virulence factors and biofilm-forming ability (32–35)	22%—S-biopsy
*Talaromyces*	Opportunistic systemic pathogen capable of causing disseminated infection that may mimic other diseases, particularly in immunocompromised patients (36, 37)	35.1%—P-blood
*Alternaria*	Phaeohyphomycosis in immunocompromised patients, cutaneous and pulmonary infection reported (8, 26, 38–40)	1.22%—P-blood
*Rhodotorula*	Bloodstream infections, especially in patients with central venous catheters, as well as central nervous system, ocular and other less frequent infections (41, 42)	26.5%—P-BAL 3.24%—S-BAL
*Cutaneotrichosporon*	Opportunistic yeasts causing subcutaneous and airway infections in susceptible patients (43, 44)	9.15%—P-BAL

### 4.1 Limitations of the study

It is challenging to determine the susceptibility of the macroorganism to fungal infections from a true sarcoidosis onset, independent of immunosuppressants. As previously described in the “Materials and methods” section, all our patients were naïve, i.e., newly diagnosed and untreated with drugs. Most of the limitations of our study were discussed in the Results and Discussion sections. These deep limitations make our study a valuable step in fundamental research into a disease whose etiology has been unknown for more than 150 years.

### 4.2 Conclusions

The relationship between fungal agents and sarcoidosis is complex and not yet fully understood. Our findings demonstrate that both sample type and processing method strongly influence the observed structure of fungal communities, with cultured samples showing reduced diversity dominated by fast-growing taxa, while non-cultured, biopsy, and respiratory-derived materials revealed broader and more heterogeneous profiles. These differences underscore the importance of methodological considerations when characterizing the human mycobiome and highlight the potential for background environmental signal to confound interpretation in control samples.

The detected fungal diversity relates to environmental and endogenous origins in blood and tissue samples. Distinct taxa reflect niche-specific colonization or translocation. The prominence of opportunistic and immunomodulatory genera such as *Candida, Saccharomyces*, and *Penicillium* suggests that these communities may actively shape host immune responses and contribute to the infectious etiology, immunopathogenesis or progression of sarcoidosis. Taken together, this work not only emphasizes the critical role of sampling strategy in microbiome studies but also indicates that future research should focus on the functional activity, host interactions, and temporal dynamics of fungi to better clarify their role in disease onset, persistence, and resolution. Treatment with antifungal drugs may be useful, at least in certain cases of sarcoidosis (29).

## Data Availability

The analyzed datasets for this study can be found in the GitHub Repository https://github.com/Rhinrei/sarcoidosis_microbiome.
